# Direct Lytic Agents: Novel, Rapidly Acting Potential Antimicrobial Treatment Modalities for Systemic Use in the Era of Rising Antibiotic Resistance

**DOI:** 10.3389/fmicb.2022.841905

**Published:** 2022-03-03

**Authors:** Raymond Schuch, Cara Cassino, Xavier Vila-Farres

**Affiliations:** ContraFect Corporation, Yonkers, NY, United States

**Keywords:** lysin, cell wall hydrolase, biologic, antimicrobial, antibiotic resistance, peptides

## Abstract

Direct lytic agents (DLAs) are novel antimicrobial compounds with unique mechanisms of action based on rapid cell wall destabilization and bacteriolysis. DLAs include two classes of purified polypeptides—lysins (peptidoglycan hydrolase enzymes) and amurins (outer membrane targeting peptides). Their intended use is to kill bacteria in a manner that is complimentary to and synergistic with traditional antibiotics without selection for DLA resistance. Lysins were originally described as having activity against Gram-positive pathogens and of those, exebacase, is the first to have advanced into Phase 3 of clinical development. Recently, both engineered and native DLAs have now been described with potent bactericidal activity against a range of Gram-negative pathogens, including multidrug-resistant (MDR) and extensively drug-resistant (XDR) *Pseudomonas aeruginosa*, *Klebsiella pneumoniae*, and *Acinetobacter baumannii*. Importantly, novel DLAs targeting Gram-negatives, including the lysin CF-370 and the amurin peptides, are active in biological matrices (blood/serum) and, as such, offer promise for therapeutic use as systemically administered agents for the treatment of life-threatening invasive infections. In this review, DLAs are discussed as potential new classes of antimicrobial biologics that can be used to treat serious systemic infections.

## Introduction

**Figure 1:**
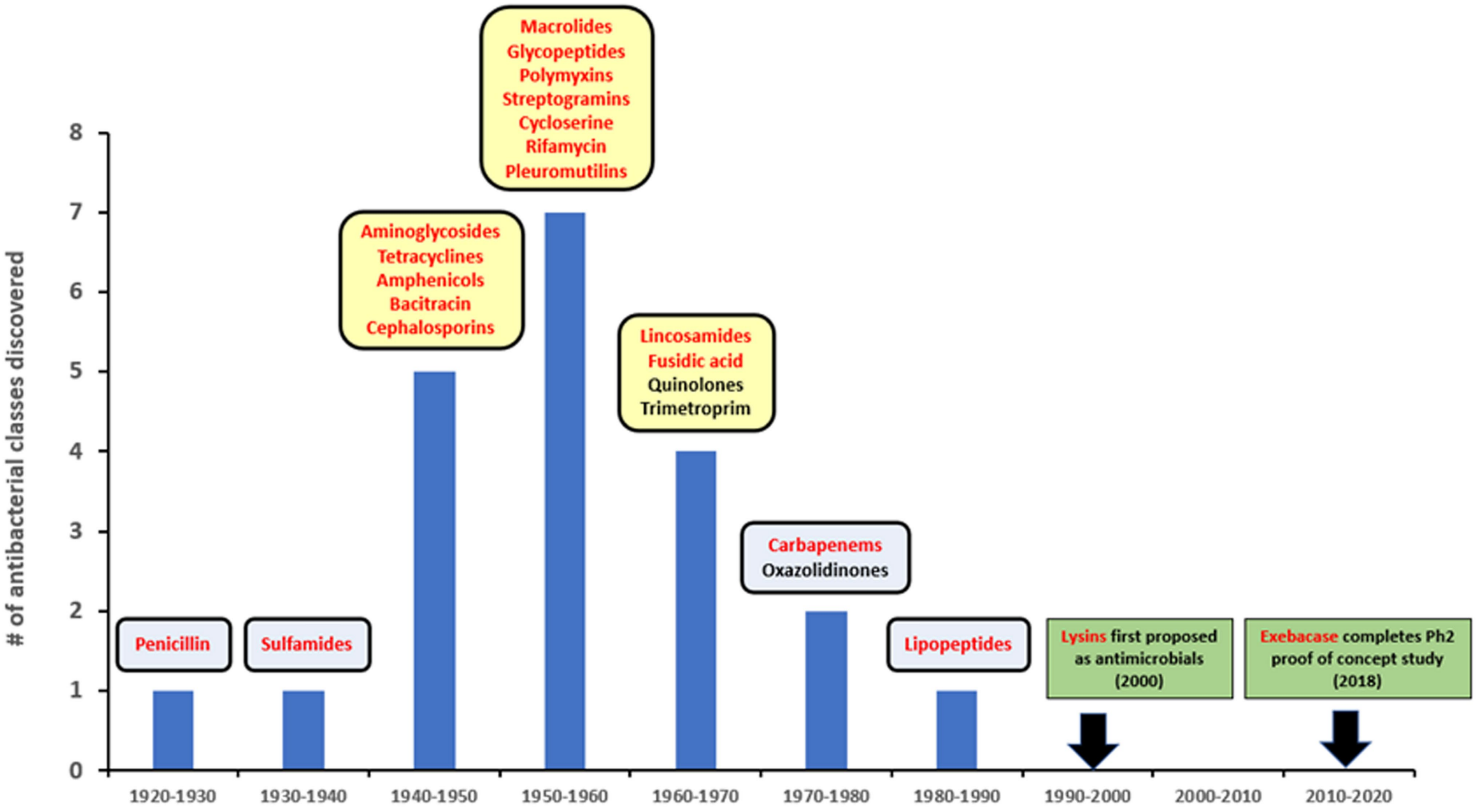
Lysins in the timeline of discovery for major antibiotic classes. Yellow squares represent golden age of antibiotics. Red font is used to show compounds with a natural origin.

Since the introduction of penicillin, new antibiotics have been primarily identified from natural sources, often bacteria and fungi, or as derivatives of pre-existing compounds (Silver, 2011). There have been no novel antibacterial drugs with distinct targets and/or mechanisms of action introduced over the last 30+ years (Figure 1), despite technological progress and extensive high-throughput screening of natural and synthetic product libraries (Singh et al., 2017; Travis et al., 2018; Serafim et al., 2020). Compounding this problem, many large pharmaceutical firms have now abandoned the anti-infective sector, leaving smaller companies and funding bodies to fill the gap (Cole, 2014). Emerging resistance to traditional antibiotics coupled with this lack of new medical modalities to treat infections caused by multidrug-resistant (MDR) and extensively drug-resistant (XDR) pathogens, pose increasingly concerning potential public health threats (WHO, 2019; Boucher, 2020).

## Direct Lytic Agents: Novel Antibacterial Proteins and Peptides

While continued advances in genomics, bioinformatics, and structural biology still offer the promise of finding new small molecule antibiotics, altogether new chemotypes with innovative antimicrobial strategies are providing advanced leads that may eventually become the next generation of compounds in clinical use. Of particular note is a novel family of antimicrobials, recently referred to as “Direct Lytic Agents (DLAs)” by ContraFect Corporation (Sauve et al., 2021; Watson et al., 2021a,b). The DLA family consists of highly purified proteins and peptides, originally derived from the lytic systems of bacteriophage and which share a set of common microbiologic attributes and mode of action based on the direct lysis of target pathogens (Wittekind and Schuch, 2016). There are, thus far, two distinct classes of DLAs, including the lysins and amurin peptides, which are differentiated from each other and from antibiotics with respect to structure and activity.

Herein we review DLAs that are active in blood matrices and can be administered systemically, focusing primarily on the antistaphylococcal lysin exebacase, which is in Phase 3 clinical development, and then on recently described engineered lysins and amurin peptides that have demonstrated *in vitro*, *ex vivo*, or *in vivo* activity against Gram-negative pathogens. The identification of DLAs that can target Gram-negative bacteria which are amenable to systemic administration represents a major advance in the field, addressing unmet clinical needs by offering potential treatments against antibiotic-resistant pathogens that cause life-threatening infections for which there are no or limited therapeutic options.

## Lysins: A Class of DLAs

The lysin class of DLAs encompass a large and structurally/functionally diverse family of cell wall hydrolytic enzymes (Fischetti et al., 2006; Nelson et al., 2012). There are five functional types of lysins: N-acetylmuramidases (lysozymes), endo-β-N-acetylglucosaminidases, and lytic transglycosylases, cleaving the amino sugar moieties of peptidoglycan; N-acetyl-muramoyl-L-alanine amidases, cleaving the amide bond between N-acetylmuramic acid and L-alanine in the stem peptide; and endopeptidases, cleaving within the peptide structure of peptidoglycan (Broendum et al., 2018; Vermassen et al., 2019). Lysins targeting Gram-positive bacteria (and to a lesser extent Gram-negative bacteria) typically also require cell wall-binding domains that direct catalytic activities to the cell wall *via* binding to either peptidoglycan or secondary cell wall components (Ganguly et al., 2013; Schuch et al., 2013; Bustamante et al., 2017; Santos et al., 2019; King et al., 2021). Binding domains direct rapid attachment with high equilibrium affinities (Schmelcher et al., 2010) and, depending on the binding epitope, may direct catalytic activities in either a species-specific (Schmelcher and Loessner, 2014) or more widespread (Schuch et al., 2019; Watson et al., 2019a) manner. Interestingly, the catalytic and cell wall-binding modules of lysins can be separable and swappable, providing ample opportunities for engineering (Gerstmans et al., 2018).

**Figure 2:**
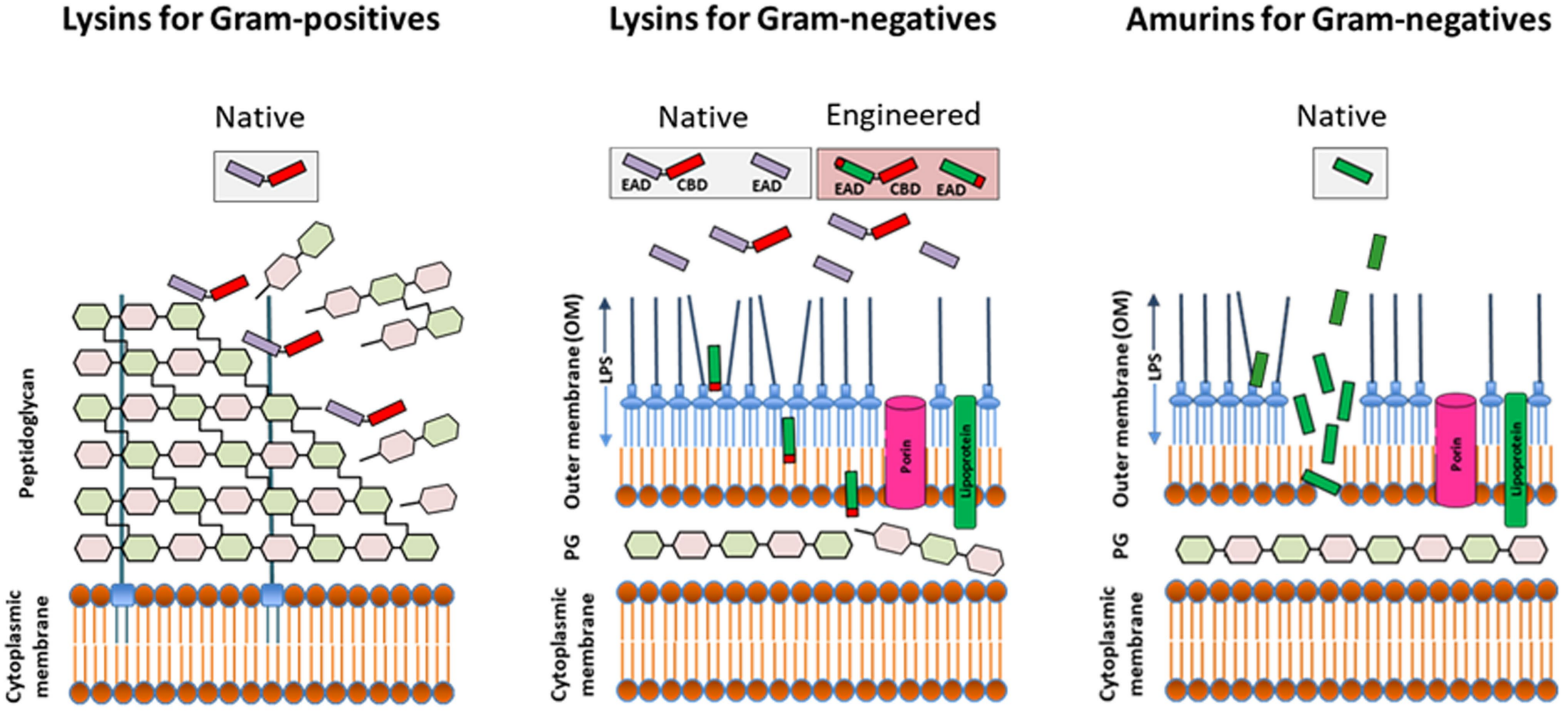
Direct lytic activity of purified (recombinant) lysins and synthesized amurin peptides applied exogenously to susceptible pathogens. The lysin class of Direct lytic agents (DLAs) consist of cell wall hydrolases targeting the surface exposed peptidoglycan of Gram-positives and the internal peptidoglycan layer of Gram-negatives. While native (unmodified) lysins are effective against Gram-positives, they require engineering to displace or disrupt the outer membrane (OM) and facilitate access to subjacent peptidoglycan in Gram-negatives. The amurin peptide class of DLAs has a non-murolytic MOA based on disruption of the OM. EAD, enzymatically active domain; CBD, cell wall-binding domain; PG, peptidoglycan; and LPS, lipopolysaccharide.

The use of lysins as non-antibiotic antimicrobials was first proposed in the early 2000s (Figure 1), based on studies showing that recombinantly produced and purified lysins can be applied exogenously to Gram-positive bacteria to elicit peptidoglycan hydrolysis and osmotic lysis (Loeffler et al., 2001; Nelson et al., 2001; Schuch et al., 2002; Fischetti et al., 2006). The ability of purified lysin to rapidly kill Gram-positive pathogens (as demonstrated in Figure 2) provided the foundation for their further development as powerful new antibacterial therapeutics.

Lysins with *in vitro* bacteriolytic activities against an array of Gram-positive organisms, including staphylococci, streptococci, *Clostridium* spp., *Paenibacillus larvae*, *Bacillus anthracis*, *Listeria monocytogenes* and others, have been described and reviewed in the literature (Schuch et al., 2002; Nelson et al., 2012; Oliveira et al., 2015; Fischetti, 2017; Haddad Kashani et al., 2018; Murray et al., 2021; Sekiya et al., 2021). A limited number of these have advanced toward the clinic. Micreos Human Health (Bilthoven, Netherlands) recently reported results from open label, interventional, clinical study in South Africa, examining the safety and efficacy of a cream containing the antistaphylococcal lysin Endobioma™ (formerly Staphefekt™ SA.100) applied to 43 adults and children with mild to moderate atopic dermatitis, which produced a statistically and clinically significant reduction of disease severity and improved skin sensitivity and quality of life (Moreau et al., 2021). A second antistaphylococcal lysin, XZ.700, is also under development by Micreos as a topical treatment of atopic dermatitis and is currently in a phase I/IIa, single-center, randomized, double-blind, placebo-controlled, parallel treated dose-ranging study with 48 patients in The Netherlands (Murray et al., 2021). Roivant Sciences Inc. (New York, NY, United States) recently reported results from a Phase 1 study (ClinicalTrials.gov identifier NCT01855048) examining the safety, pharmacokinetics, and pharmacodynamics of single dose of LSVT-1701 (an antistaphylococcal lysin formerly called N-Rephasin® SAL200) in 32 healthy male subjects at a single center in South Korea (Wire et al., 2022). LSVT-1701 lysin was safe and well-tolerated; however, the Phase 2 trial was terminated for strategic reasons (ClinicalTrials.gov Identifier: NCT03089697).

Standing out as a potential therapeutic is the lysin exebacase (formerly *CF*-301), which is currently under development by ContraFect Corporation (Yonkers, NY, United States). Exebacase has been granted Breakthrough Therapy designation by the FDA and is the first and only lysin to advance into Phase 3 of clinical development as a systemically administered treatment to improve clinical outcomes for *Staphylococcus aureus* bacteremia, including right-sided endocarditis (ClinicalTrials.gov Identifier NCT04160468). Based on its position as a first-in-class and first-in-field compound, the discovery and development of exebacase is next described, with a focus on its hallmark *in vitro* microbiologic attributes, efficacy in animal models of infection, and results from a superiority-design, proof-of-concept Phase 2 clinical trial in the United States.

## Exebacase: A Lysin Targeting *Staphylococcus aureus*

Exebacase was initially identified as a potent antistaphylococcal lysin with d-alanyl-l-glycyl endopeptidase activity (Schuch et al., 2014; Lood et al., 2017). It has a modular structure, 26 kilodaltons in size, with an N-terminal cysteine–histidine-dependent amidohydrolases/peptidases (CHAP) domain and a C-terminal SH3b cell wall-binding domain. A rapid bacteriolytic activity of exebacase against antibiotic-resistant *S. aureus* served as the initial basis for its further development (Schuch et al., 2014).

An accurate and reproducible method for antimicrobial susceptibility testing of *S. aureus* was developed for exebacase (CLSI, 2020; Oh et al., 2021a; Traczewski et al., 2021) and used in a surveillance study of clinical methicillin-susceptible and methicillin-resistant *S. aureus* isolates (MSSA and MRSA, respectively) collected from 2015 to 2017 in the United States, Europe, and South America. The clinical isolates were inhibited at ≤1 μg/ml of exebacase with 50% and 90% of organisms (MIC_50_ and MIC_90_, respectively) inhibited at 0.5 and 1 μg/ml, respectively (Traczewski et al., 2019). Exebacase was also uniformly active against a set of 666 contemporary *S. aureus* isolates responsible for bloodstream infections in the US in 2020, with an MIC range of 0.06–1 μg/ml and with MIC_50_, MIC_90_, and modal MIC values of 0.5 μg/ml (Mendes et al., 2021). No difference in activity was observed between the MSSA and MRSA isolates. In time-kill studies, exebacase used at 1x strain-specific MIC levels rapidly reduced *S. aureus* viability by 3-log_10_ within 30 min at the MIC (Schuch et al., 2014). Synergy was observed for interactions tested with each of 12 antibiotics, where checkerboard studies with *S. aureus* isolates showed fractional inhibitory concentration index values of ≤0.5, and this synergetic effect was further confirmed in time–kill assays (Watson et al., 2020). Exebacase is also highly active against *S. aureus* in human serum, plasma, and whole blood (Indiani et al., 2019), synovial fluid (Oh et al., 2019a), and pulmonary surfactant (Swift et al., 2021).

Exebacase is furthermore active against *S. aureus* persister cells and biofilm bacteria (Schuch et al., 2017). In biofilm studies, exebacase demonstrated a minimal biofilm eradicating concentration (MBEC) of ≤0.25 μg/ml for 90% of organisms tested, including 55 MRSA strains (Schuch et al., 2017). Biofilms formed on polystyrene, glass, surgical mesh, and catheter surfaces were all susceptible to exebacase, as were biofilms treated in the context of human serum, pulmonary surfactant (±daptomycin), and synovial fluid (Schuch et al., 2017; Swift et al., 2021). Bacteria in biofilms are highly recalcitrant to antibiotic therapy and novel strategies that target biofilms are highly desirable (de la Fuente-Nunez et al., 2013). Based on these findings, exebacase may have therapeutic potential as a new medical modality to treat biofilm-associated *S. aureus* infections.

Short and/or sub-MIC exposure of exebacase to *S. aureus* exerts suppressive effects on both growth and virulence traits (Oh et al., 2019b). Mean post-antibiotic effect (PAE), post-antibiotic sub-MIC effect (PA-SME), and sub-MIC effect (SME) values up to 4.8, 9.3, and 9.8 h, respectively, were observed in human serum, and growth inhibition was extended by up to 6 h in the additional presence of sub-MIC of daptomycin. Exposures to exebacase at sub-MIC levels as low as 0.001x MIC resulted in aberrant cell wall ultrastructure, increased membrane permeability, dissipation of membrane potential, and the inhibition of virulence phenotypes, including agglutination and biofilm formation. These findings suggest that the bactericidal activity of exebacase may be complemented by antivirulence functions at concentrations well below the MIC (Oh et al., 2019b).

Importantly, exebacase exhibits a low propensity for resistance development and the ability to suppress resistance development of other antibiotics when used in combination (Schuch et al., 2014; Oh and Schuch, 2017; Oh et al., 2018). Serial passage of exebacase alone resulted in only up to 2-fold increases in MIC, and when used at sub-MIC concentrations in addition to daptomycin, vancomycin, and oxacillin, exebacase suppressed the development of resistance for those antibiotics. Resistance development by spontaneous mutation seems to be a rare event for lysins, which may be attributed to the essential and conserved nature of peptidoglycan bonds that are the targets for these lysins (Fischetti et al., 2006). Resistance spread by horizontal transfer between intrinsically resistant and susceptible species, while unlikely, cannot be ruled out (Grishin et al., 2020).

The rapid bactericidal activity (Schuch et al., 2014), synergy with antibiotics (Schuch et al., 2014; Wittekind and Schuch, 2016; Watson et al., 2020), antibiofilm activity (Schuch et al., 2014, 2017), extended post-antibiotic effect (Oh et al., 2019b), and low propensity for resistance development (Schuch et al., 2014; Oh and Schuch, 2017; Oh et al., 2018) differentiate exebacase from traditional small molecule antibiotics and support further development of this DLA.

## Exebacase: Efficacy in Animal Models

Results from *in vitro* synergy studies with exebacase and antibiotics, correlate with data from pre-clinical pharmacology studies showing potent *in vivo* activity for exebacase used in addition to conventional antistaphylococcal antibiotics including daptomycin, vancomycin, and oxacillin.

In a model of infective endocarditis infection with MRSA in rabbits, the addition of exebacase to either daptomycin or vancomycin resulted in significant reductions in colony-forming units (CFUs) in all target organs when compared to reductions observed with either antibiotic alone (Indiani et al., 2019). A single intravenous (i.v.) administration of exebacase as low as 0.09 mg/kg (in addition to a subhuman-equivalent dose of daptomycin) achieved a 6-log_10_-unit drop in CFUs per gram in heart valve vegetations compared to a 3-log_10_-unit drop in CFUs with daptomycin alone.

In a mouse model of MRSA bacteremia, exebacase administered i.v. in addition to daptomycin resulted in more than 70% survival, compared to less than 30% survival for daptomycin alone (Schuch et al., 2014). A single i.v. dose of exebacase in addition to daptomycin administered intraperitoneally every 12 h for 4 days also resulted in an additional decrease of more than 1.5 log_10_ CFU/gram of bone (compared to daptomycin alone) in a rat model of MRSA osteomyelitis (Karau et al., 2019). In the neutropenic murine thigh infection model, the addition of exebacase to daptomycin resulted in a 1.03 ± 0.72 log_10_ CFU/thigh reduction, whereas daptomycin alone resulted in growth of 0.39 ± 1.19 log_10_ CFU/thigh (Asempa et al., 2020). Finally, in a murine pneumonia model, exebacase used in addition to daptomycin resulted in 70% survival at 14 days, whereas treatment with daptomycin alone yielded no survivors (Swift et al., 2021).

## Exebacase: First DLA to Enter Clinical Trials

As a first-in-class antimicrobial agent with the potential to improve clinical outcomes of *S. aureus* infections, exebacase completed a superiority-design, proof-of-concept Phase 2 clinical trial (Fowler et al., 2020). This randomized, double-blind, placebo-controlled study assessed the clinical response rates of patients with *S. aureus* bacteremia including endocarditis treated with a single i.v. infusion of exebacase in addition to standard-of-care antibiotics (SoCA) versus SoCA treatment.

Overall, 70.4% of patients in the exebacase and SoCA group and 60.0% of patients who received SoCA alone, were clinical responders at Day 14 (value of *p* = 0.3137). Importantly, in the pre-specified MRSA subgroup, the clinical responder rate at Day 14 was 42.8 percentage points higher in the exebacase-treated group compared with the SoCA alone group (responder rates of 74.1% vs. 31.3%, respectively; *ad hoc* value of *p* = 0.0101). Furthermore, a 21-percentage point lower 30-day all-cause mortality rate was observed among patients carrying MRSA that received exebacase with SoCA compared with those that received SoCA alone (3.7% vs. 25.0%, *p* = 0.0556). Among MRSA patients in the United States, the median length of stay was 6 days for patients treated with exebacase and SoCA, compared to 10 days for patients treated with antibiotics alone; a trend toward lower 30-day all-cause readmission rates (48% lower) was also observed in the exebacase-treated group compared with antibiotics alone. Exebacase was, furthermore, generally safe and well-tolerated, with adverse events consistent with those expected in critically ill, hospitalized patients with potentially life-threatening *S. aureus* bloodstream infection, including endocarditis and/or underlying comorbid conditions.

Based on the results of the Phase 2 study, exebacase has progressed into Phase 3 of clinical development for the treatment of *S. aureus* bacteremia including right-sided endocarditis and was granted Breakthrough Therapy designation for this indication by the FDA.

## CF-370: First Lysin That Can Be Administered Systemically to Target Gram-Negative Pathogens

Unlike lysins targeting the surface exposed peptidoglycan of Gram-positive bacteria, exogenously applied lysins targeting Gram-negatives must first translocate across or permeabilize the outer membrane (OM) to reach their target peptidoglycan (Figure 2). The OM is a very effective barrier to bulky molecules with molecular masses of more than ∼600 Da, including lysins (Delcour, 2009; Silhavy et al., 2010). Thus, for most lysins to be active against Gram-negative bacteria, the use of either high hydrostatic pressures to transiently permeabilize the OM (Nakimbugwe et al., 2006; Briers et al., 2008) or the simultaneous addition of membrane destabilizing agents like poly-L-lysine, polymyxin B, ethylenediaminetetraacetic acid (EDTA), organic acids, or the essential oil carvacrol were originally required (Briers et al., 2011; Legotsky et al., 2014; Oliveira et al., 2014; Shavrina et al., 2016; Antonova et al., 2019; Ciepluch et al., 2019).

The long-held belief that the OM barrier precludes use of purified lysins against Gram-negative bacteria was first dispelled by the description of several lysins with “intrinsic” OM penetrating activity. Carboxy-terminal amphipathic α-helices within these lysins mediated translocation across the OM to reach the subjacent peptidoglycan with subsequent hydrolysis and cell killing (Lood et al., 2015; Larpin et al., 2018; Raz et al., 2019; Vasina et al., 2021; Vazquez et al., 2021). The intrinsic activity of these lysins is, however, restricted to low osmotic strength buffers and environments lacking physiological concentrations of NaCl and divalent cations, or human blood matrices, thus precluding, systemic administration and restricting their activity *in vivo* to topical use.

Further development of lysins targeting Gram-negative pathogens exploited the modular nature of such enzymes and the capability to append functional domains, in particular antimicrobial peptides and OM permeabilizing peptides (Briers et al., 2014a,b; Sauve et al., 2018, 2019). Engineered lysin CF-370 is distinguished here as it is the first Gram-negative lysin reported active in human serum and, thus potentially amenable for systemic administration. CF-370 is active with MIC_90_ values of 0.5–4 μg/ml against strain sets that include MDR and XDR *P. aeruginosa*, *K. pneumoniae*, *Escherichia coli*, *A. baumannii*, *Enterobacter cloacae*, and *Stenotrophomonas maltophilia* (Sauve et al., 2018, 2019; Watson et al., 2019b, 2021a,b; Oh et al., 2021b). CF-370 exhibits all of the microbiologic attributes associated with lysins targeting Gram-positive bacteria, including a rapid bactericidal effect against sensitive organisms, antibiofilm activity, synergy with a range of antibiotics, and the capacity to resensitize antibiotic-resistant bacteria (Watson et al., 2019b, 2021a,b).

Two pre-clinical pharmacology studies provided *in vivo* proof-of-concept efficacy data supporting the systemic administration of CF-370. In a model of acute *P. aeruginosa* pneumonia in rabbits, i.v. administration of CF-370 as a single dose (at 3 and 10 mg/kg) in addition to meropenem (20 mg/kg, q8h) achieved a 3-log_10_-CFU reduction per gram of lung compared to a 1-log_10_ reduction with either meropenem or CF-370 alone (Lehoux et al., 2021a). CF-370 also limited proliferation in secondary organs, as significant reduced bacterial counts were observed in kidney and spleen when used alone or in addition to meropenem. In a rabbit model of right-sided infective endocarditis caused by *P. aeruginosa*, 3 daily i.v. doses of CF-370 in addition to meropenem resulted in a 2-log_10_ CFU reduction per gram of heart vegetation compared to meropenem alone (Lehoux et al., 2021b). Up to 2.2-log_10_ were also observed in secondary organs, compared to meropenem alone. The *in vivo* efficacy observed for systemically administered CF-370 in these two models of invasive infection clearly distinguishes CF-370 from other engineered lysins which were not active in serum and for which efficacy data are limited to a skin infection model using human keratinocytes (Briers et al., 2014b), nematode and wax moth models (Briers et al., 2014b; Chen et al., 2021), an *ex vivo* pig and mouse burn wound model (Gerstmans et al., 2020; Li et al., 2021), or treatment of ear infections in dogs (Briers and Lavigne, 2015).

The *in vitro* and *in vivo* data for CF-370 is the first evidence that lysins can be engineered for i.v. delivery. This work spectrum antimicrobial provides a solid basis for further development of CF-370 (and, potentially, other engineered lysins) as a treatment for serious and life-threating infections with MDR and XDR Gram-negative pathogens.

## Amurin Peptides: New Class of DLAs Targeting Gram-Negative Pathogens

An entirely new class of DLAs were recently identified, called amurin (for “amurolytic”) peptides, with bactericidal activity against a broad range of Gram-negative ESKAPE bacteria including carbapenem-resistant *Enterobacterales* (CRE), *P. aeruginosa* (CRP), and *A. baumannii* (CRA; Sauve et al., 2021). Derived from the lytic systems of phage, the amurins consist of a related family of small alpha-helical cationic peptides, typically 40–50 amino acids in length and distinct in sequence from previously described antimicrobial peptides (Sauve et al., 2021).

As synthesized peptides, amurins demonstrate a mechanism of action based on rapid OM-permeabilization, distinct from the cell wall hydrolytic activity of lysin enzymes (Figure 2; Sauve et al., 2021). For select amurins, MIC_90_ values of ≤1 μg/ml were reported against strain sets of *P. aeruginosa*, *A. baumannii*, *K. pneumoniae*, *E. cloacae*, and *E. coli*. The amurins are furthermore active in human serum and blood and exhibit many of the hallmark microbiological attributes associated with the lysins including *in vitro* and *ex vivo* antibiofilm activity (with MBEC values ranging from 0.125 to 2 μg/ml), synergy with up to 11 different conventional antibiotics tested and a low propensity for resistance development (Oh et al., 2019c; Sauve et al., 2021).

The discovery of an entirely new class of DLAs with potent activity against MDR and XDR Gram-negative pathogens and with a mechanism of action distinct from lysins brings the promise for further expanding the armamentarium of differentiated antimicrobial agents for treating invasive life-threatening infections.

## Conclusion

In this era of diminished antibiotic susceptibility, DLAs provide new and highly differentiated antimicrobial modalities. The promise for DLAs lies in their diverse mechanisms of action and range of potent activity against both Gram-positive and Gram-negative bacteria. Key microbiological attributes shared among the DLAs are rapid bactericidal activity, antibiofilm activity, synergy with antibiotics, and a low propensity for resistance. Exebacase has provided proof-of-concept data in humans for the DLA platform, as it proceeds through clinical trials to treat life-threatening *S. aureus* bacteremia including right-sided endocarditis. CF-370 is the first DLA that can be administered systemically to treat infections caused by antibiotic-resistant Gram-negative bacteria. This enzyme was developed through protein engineering to allow new lysin specificities permeate through the OM barrier. Finally, the amurin peptides have been shown to be unique DLAs that provide differentiated mechanisms of antimicrobial action. Moving forward, still other lysins, amurin peptides, and perhaps altogether new DLAs are envisioned to reinvigorate the pipeline for new antimicrobial agents to satisfy the unmet medical need of treating infections caused by multidrug-resistant bacteria.

## Author Contributions

RS, CC, and XV-F conceptualized and wrote the manuscript. All authors contributed to the article and approved the submitted version.

## Funding

ContraFect receives funding from CARB-X, which is sponsored by the Cooperative Agreement Number IDSEP160030 from ASPR/BARDA and by an award from Wellcome Trust.

## Conflict of Interest

RS, CC, and XV-F are employees of ContraFect Corporation.

## Publisher’s Note

All claims expressed in this article are solely those of the authors and do not necessarily represent those of their affiliated organizations, or those of the publisher, the editors and the reviewers. Any product that may be evaluated in this article, or claim that may be made by its manufacturer, is not guaranteed or endorsed by the publisher.
